# The Predictive Value of The Hemoglobin, Albumin, Lymphocyte, and Platelet (HALP) Score for One-Month Prognosis and First-Year Mortality in Patients Undergoing Transcatheter Aortic Valve Implantation

**DOI:** 10.21470/1678-9741-2024-0300

**Published:** 2026-09-10

**Authors:** Güney Erdoğan, Mustafa Yenercağ, Serkan Sivri, Melisa Uçar, Halil İbrahim Kökçü, Enes Kaya, Yüksel Erata, Mertcan Erzincan, Seçkin Satılmış, Orhan İnce, İrfan Şahin

**Affiliations:** 1 Cardiology Clinic, Samsun Training and Research Hospital, University of Health Sciences Turkey, Samsun, Turkey; 2 Department of Cardiology, Samsun University, Samsun, Turkey; 3 Department of Cardiology, Kafkas University, Kars, Turkey; 4 Department of Emergency, Bafra State Hospital, Samsun, Turkey; 5 Department of Cardiology, Acibadem Atakent University Hospital, Istanbul, Turkey; 6 Department of Cardiology, Bağcılar Training and Research Hospital, University of Health Sciences, Istanbul, Turkey

**Keywords:** HALP Score, TAVI, One-Month Prognosis, First-Year Mortality.

## Abstract

**Objective:**

Transcatheter aortic valve implantation (TAVI) has become a viable option for patients with severe aortic valve stenosis across a broad range of surgical risk in recent years. The Hemoglobin, Albumin, Lymphocyte, and Platelet (HALP) score, a novel indicator of malnutrition and inflammation, has been discovered to be inversely linked with prognosis in several cancer types. The purpose of this study is to examine the performance of the HALP score in predicting one-month major adverse cardiac events and one-year mortality in patients treated with TAVI.

**Methods:**

This study included 395 consecutive patients treated with TAVI, separated into two groups based on their HALP scores: low and high. Our study's primary endpoint was all-cause death within a year after discharge following the TAVI operation. The secondary endpoint was a composite endpoint that included periprocedural complications and events in one month.

**Results:**

Patients with a low median HALP score had a higher risk of one-month composite events and one-month death. In multivariate analysis, chronic kidney disease (odds ratio [OR]: 4.67, 95% confidence interval [CI]: 2.36 - 9.25, P < 0.001) and HALP score < 3.4 (OR: 1.235, 95% CI: 1.091 - 1.397, P < 0.001) were independent predictors of first-year mortality. Kaplan-Meier analysis for computing cumulative survival found that patients with low HALP scores based on a receiver operating characteristic curve cutoff of 3.4 had increased mortality rates throughout short-term follow-up.

**Conclusion:**

The HALP score may be a significant independent predictor of short-term prognosis and mortality in patients treated with TAVI, beyond conventional risk-scoring tools for better disease management.

## INTRODUCTION

**Table:** 

Abbreviations, Acronyms & Symbols				
AF	= Atrial fibrillation		LVEF	= Left ventricular ejection fraction
AKI	= Acute kidney injury		MACE	= Major adverse cardiac events
AR	= Aortic regurgitation		OR	= Odds ratio
AS	= Aortic stenosis		PAD	= Peripheral artery disease
BMI	= Body mass index		PCI	= Percutaneous coronary intervention
CAD	= Coronary artery disease		PROM	= Predicted Risk of Mortality
CEP	= Composite endpoint		ROC	= Receiver operating characteristic
CI	= Confidence interval		SII	= Systemic immune-inflammation index
CKD	= Chronic kidney disease		STEMI	= ST-segment elevation myocardial infarction
COPD	= Chronic obstructive pulmonary disease		STS	= Society of Thoracic Surgeons
DM	= Diabetes mellitus		TAVI	= Transcatheter aortic valve implantation
EF	= Ejection fraction		TAVR	= Transcatheter aortic valve replacement
HALP	= Hemoglobin, albumin, lymphocyte, and platelet		VARC-3	= Valve Academic Research Consortium 3

Over the past decade, transcatheter aortic valve implantation (TAVI) has become a widely accepted treatment for patients with severe aortic stenosis (AS) who are at increased surgical risk. Previous studies have demonstrated comparable twoand five-year outcomes for TAVI and surgical aortic valve replacement in lowand intermediate-risk patients, respectively^[^1^-^3^]^. In one trial, TAVI even showed superiority over surgery at two years in low-risk patients^[^4^]^. In recent years, severe AS has also been recognized as an inflammatory disease, with a pathophysiologic profile involving lipoprotein accumulation, calcification, and chronic inflammation leading to atherosclerosis^[^5^]^. Meanwhile, cancer cachexia, a complex, multifactorial syndrome characterized by progressive skeletal muscle loss, has been linked to anemia, hypoalbuminemia, and malnutrition in patients with cancer^[^6^]^. The hemoglobin, albumin, lymphocyte, and platelet (HALP) score, which reflects nutritional and systemic inflammatory status, is particularly used in older adult populations. The HALP score is calculated as follows: hemoglobin (g/L) × albumin (g/L) × lymphocyte count (10^9^/L) / platelet count (10^9^/L)^[^7^]^. It was originally developed by Chen et al.^[^7^]^ as a prognostic tool in gastric carcinoma and has since been studied in other cancers, in which it has consistently demonstrated an inverse relationship with clinical outcomes^[^8^-^10^]^. Specifically, lower HALP scores, because of low hemoglobin, albumin, and lymphocyte counts and high platelet counts, have been associated with a worse prognosis.

To date, no study has investigated the role of the HALP score in predicting short-term outcomes in patients undergoing TAVI. Therefore, the current study aimed to investigate whether the HALP score, a novel immunomalnutrition marker in various cancers, can predict major adverse cardiac events (MACE) within one month, including periprocedural complications, and one-year all-cause mortality in patients treated with TAVI.

## METHODS

### Study Population

This was a single-center, retrospective cohort study conducted at the Cardiology Clinic of Samsun Research and Training Hospital. A total of 413 patients who were eligible for TAVI based on standard clinical indications were registered between January 2018 and December 2023. The exclusion criteria were as follows: (1) history of malignancy, (2) end-stage liver disease, (3) chronic inflammatory disease, (4) autoimmune disorders, and (5) active infection. After applying these criteria, the final study population consisted of 392 patients.

Clinical, demographic, echocardiographic, and procedural data were retrieved from the hospital’s electronic medical records. Patient eligibility was assessed using the Society of Thoracic Surgeons (STS) score, which estimates perioperative or short-term mortality. A heart team assessed patients’ suitability for intervention. The choice between surgical aortic valve replacement and TAVI was based on the STS Predicted Risk of Mortality (PROM) score. Patients with an STS PROM score > 4%, indicating increased surgical risk, were included in this study.

The study was conducted in accordance with the principles outlined in the Declaration of Helsinki. Written informed consent was obtained from all patients, and the study protocol was approved by the local research ethics committee.

### Hemoglobin, Albumin, Lymphocyte, and Platelet (HALP) Score

Whole blood samples were collected preoperatively from the antecubital vein in all patients. Hematologic and biochemical parameters were measured using automated analyzers (Beckman Coulter Hematology Analyzer LH780 and Chemistry Analyzer AU680). Five preoperative hematologic indices - hemoglobin, albumin, lymphocyte count, neutrophil count, and platelet count - were used to calculate the HALP score as follows:


$$\text{HALP}=\text{hemoglobin (g/L)}\times \text{albumin (g/L)}\times \text{lymphocyte count}(10^{9}/L) /\text{platelet count}(10^{9}/L)$$

### Study Endpoints

Periprocedural complications and clinical endpoints were defined according to the Valve Academic Research Consortium 3 (VARC-3) criteria^[^11^]^. Periprocedural complications included all-cause mortality; vascular access-related complications; cardiac structural complications; conduction disturbances necessitating permanent pacemaker implantation; overt bleeding requiring blood transfusion, classified according to the Bleeding Academic Research Consortium criteria^[^12^]^; neurologic events occurring within 30 days; and acute kidney injury (AKI) occurring within seven days of the index procedure.

The primary endpoint of the study was all-cause mortality within one year of discharge following the TAVI. The secondary endpoint was a composite one-month outcome comprising all-cause mortality, stroke, VARC type 2 - 4 bleeding, major vascular complications, access-related or cardiac structural complications, stage 3 - 4 AKI, moderate to severe aortic regurgitation, new permanent pacemaker implantation, and any surgery or intervention related to TAVI within 30 days. Short-term mortality and MACE were verified through a comprehensive review of the hospital’s electronic medical records and a state mortality database.

### Statistical Analysis

All statistical analyses were performed using IBM SPSS Statistics for Windows, version 26 (IBM Corp., Armonk, N.Y., USA). Categorical variables were reported as counts and percentages. Continuous variables were assessed for normality using the Kolmogorov-Smirnov test and reported as either mean (standard deviation) or median (interquartile range), depending on data distribution. Categorical variables were compared using the Pearson’s χ^^2^^ test or χ^^2^^ test with Fisher’s exact test. Continuous variables were compared using the Student’s *t*-test or the Mann-Whitney U test. Receiver operating characteristic (ROC) analysis was performed to assess the sensitivity and specificity of the HALP score at cutoff values, with 95% confidence interval (CI) reported. Logistic regression analysis was used to identify predictors of one-year mortality. Variables with *P* < 0.25 in univariable analysis were included in the multivariable regression model. A two-tailed *P* < 0.05 was considered statistically significant. Kaplan-Meier survival curves were generated to evaluate cumulative survival, stratified by HALP score groups based on an ROC-determined cutoff value.

## RESULTS

### Baseline Characteristics

Table 1 provides a brief overview of the baseline characteristics of the study cohort (N = 392), stratified by one-year mortality. The one-year mortality rate was 11.4% (n = 45). Patients who died within one year had a significantly higher median STS score (*P* = 0.005), a higher prevalence of chronic kidney disease (CKD) and diabetes mellitus (DM) (*P* < 0.001 and *P* = 0.018, respectively), and a lower median HALP score, compared with survivors. Conversely, there were no significant differences between groups in terms of mean age; median body mass index; ejection fraction; sex; or the presence of coronary artery disease, hypertension, stroke, peripheral artery disease, chronic obstructive pulmonary disease, and atrial fibrillation. Additionally, the proportions of patients with stage 3 - 4 AKI and VARC-3 bleeding stages 2 - 3 were comparable between groups.

**Table 1:** Baseline characteristics of patients.

Variables	Total population	Mortality	Mortality	*P*-value
	(n = 392)	1^st^ year (-)	1^st^ year (+)	
		(n = 347)	(n = 45)	
Age (years)	78.72 ± 6.96	78.72 ± 6.93	78.72 ± 7.31	0.997
Female sex, n (%)	211 (53.8)	187 (53.9)	24 (53.3)	0.944
BMI (kg/m^2^)	26.32 [22.68 - 30.99]	26.39 [22.84 - 30.48]	25.39 [21.91 - 38.13]	0.812
STS score	10.1 [6.83 - 13.7]	10 [6.8 - 11.06]	10.9 [7.85 - 16.38]	0.005
CKD, n (%)	91 (23.2)	67 (19.3)	24 (53.3)	< 0.001
CAD, n (%)	134 (34.2)	121 (34.9)	13 (28.9)	0.426
DM, n (%)	205 (52.3)	174 (50.1)	31 (68.9)	0.018
Hypertension, n (%)	371 (94.6)	330 (95.1)	41 (91.1)	0.263
Stroke, n (%)	19 (4.8)	16 (4.6)	3 (6.7)	0.468
PAD, n (%)	169 (43.1)	150 (43.2)	19 (42.2)	0.898
COPD, n (%)	291 (74.2)	259 (74.6)	32 (71.1)	0,611
AF, n (%)	185 (47.2)	163 (47)	22 (48.9)	0.809
EF (%)	55 [45 - 60]	55 [45 - 60]	60 [45-60]	0.419
AKI stage 3 - 4	11 (2.8)	8 (2.3)	3 (6.7)	0.121
VARC3 bleeding stage 2 - 4	30 (7.7)	25 (7.2)	5 (11.1)	0.368
HALP score	2.91 [2.02 - 4.38]	4.37 [2.52 - 6.41]	2.85 [2.01 - 4.02]	0.001

AF=atrial fibrillation; AKI=acute kidney injury; BMI=body mass index; CAD=coronary artery disease; CKD=chronic kidney disease; COPD=chronic obstructive pulmonary disease; DM=diabetes mellitus; EF=ejection fraction; HALP=hemoglobin, albumin, lymphocyte, and platelet; PAD=peripheral artery disease; STS=Society of Thoracic Surgeons; VARC=Valve Academic Research Consortium

### One-Month Composite Endpoints

As shown in Table 2, patients were compared using median HALP scores of the one-month composite endpoints. The one-month incidences of composite events and mortality were 28.1% and 3.8%, respectively, in the overall TAVI cohort. Patients with lower median HALP scores had a higher risk of stroke, VARC-3 type 2 - 4 bleeding, major vascular complications, access-related or cardiac structural complications, stage 3 - 4 AKI, and new permanent pacemaker implantation, compared to those with higher median HALP scores.

**Table 2:** Composite endpoints after transcatheter aortic valve replacement in one month according to HALP score.

	Event (+) n (%)	Event (-) median HALP score	Event (+) median HALP score	*P*-value
CEP	110 (28.1)	3.16 [2.68 - 5.23]	2.77 [1.96 - 4.04]	< 0.001
Mortality	15 (3.8)	5.88 [3.04 - 7.17]	2.96 [2.08 - 4.09]	< 0.001
Stroke	6 (1.5)	3.81 [3.47 - 5.92]	2.96 [2.08 - 4.35]	0.09
VARC type 2 - 4 bleeding	30 (7.7)	4.65 [2.96 - 6.68]	2.94 [2.02 - 4.06]	< 0.001
Major vascular, access-related, or cardiac structural complication	26 (6.6)	3.74 [2.87 - 5.13]	2.96 [2.04 - 4.11]	0.039
AKI stage 3 - 4	11 (2.8)	2.96 [2.95 - 7.93]	2.96 [2.08 - 4.22]	0.037
Moderate-severe AR	15 (3.8)	2.96 [2.1 - 4.38]	2.68 [2.38 - 3.7]	0.672
New permanent pacemaker	26 (6.6)	3.32 [2.89 - 5.18]	2.96 [2.04 - 4.22]	0.026
Surgery or intervention related to TAVR	12 (3.1)	3.03 [2.82 - 5.25]	2.96 [2.09 - 4.22]	0.299

A two-tailed *P*-value < 0.05 was accepted as statistically significant

AKI=acute kidney injury; AR=aortic regurgitation; CEP=composite endpoint; HALP=hemoglobin, albumin, lymphocyte, and platelet; TAVR=transcatheter aortic valve replacement; VARC=Valve Academic Research Consortium

### Univariable and Multivariable Analyses

To identify predictors of one-year mortality, both univariable and multivariable logistic regression analyses were performed (Table 3). In univariable analysis, the following variables were significantly associated with one-year mortality: CKD (odds ratio [OR], 4.476; 95% CI, 2.51 - 9.089; *P* < 0.001), DM (OR, 2.202; 95% CI, 1.132 - 4.282; *P* = .02), STS score (OR, 1.1; 95% CI, 1.022 - 1.184; *P* = .012), and HALP score (OR, 1.242; 95% CI, 1.103 - 1.399; *P* < 0.001). In multivariable analysis, only CKD (OR, 4.67; 95% CI, 2.36 - 9.25; *P* < .001) and HALP score (OR, 1.235; 95% CI, 1.091 - 1.397; *P* < .001) remained independent predictors of one-year mortality.

**Table 3:** Mortality predictors within first year of transcatheter aortic valve replacement.

Variables	Univariable OR	*P*-value	Multivariable OR	*P*-value
	(95% CI)		(95% CI)	
Age	1.0 (0.956 - 1.046)	0.997		
Male sex	0.978 (0.525 - 1.822)	0.944		
CKD	4.776 (2.51 - 9.089)	< 0.001	4.67 (2.36 - 9.25)	< 0.001
Atrial fibrillation	1.08 (0.58 - 2.01)	0.809		
CAD	0.759 (0.384 - 1.5)	0.427		
Diabetes mellitus	2.202 (1.132 - 4.282)	0.02	1.998 (0.979 - 4.08)	0.057
Hypertension	0.528 (0.169 - 1.645)	0.271		
Previous stroke	1.478 (0.413 - 5.284)	0.548		
PAD	0.96 (0.512 - 1.799)	0.798		
COPD	0.836 (0.42 - 1.665)	0.611		
BMI	1.022 (0.968 - 1.08)	0.435		
STS score	1.1 (1.022 - 1.184)	0.012	1.031 (0.946 - 1.125)	0.486
LVEF	1.006 (0.98 - 1.034)	0.648		
Low HALP score (< 3.4)	1.242 (1.103 - 1.399)	< 0.001	1.235 (1.091 - 1.397)	0.001

BMI=body mass index; CAD=coronary artery disease; CKD=chronic kidney disease; CI=confidence interval; COPD=chronic obstructive pulmonary disease; HALP=hemoglobin, albumin, lymphocyte, and platelet; LVEF=left ventricular ejection fraction; OR=odds ratio; PAD=peripheral artery disease; STS=Society of Thoracic Surgeons

### ROC and Kaplan-Meier Analysis

**Fig. 1:**
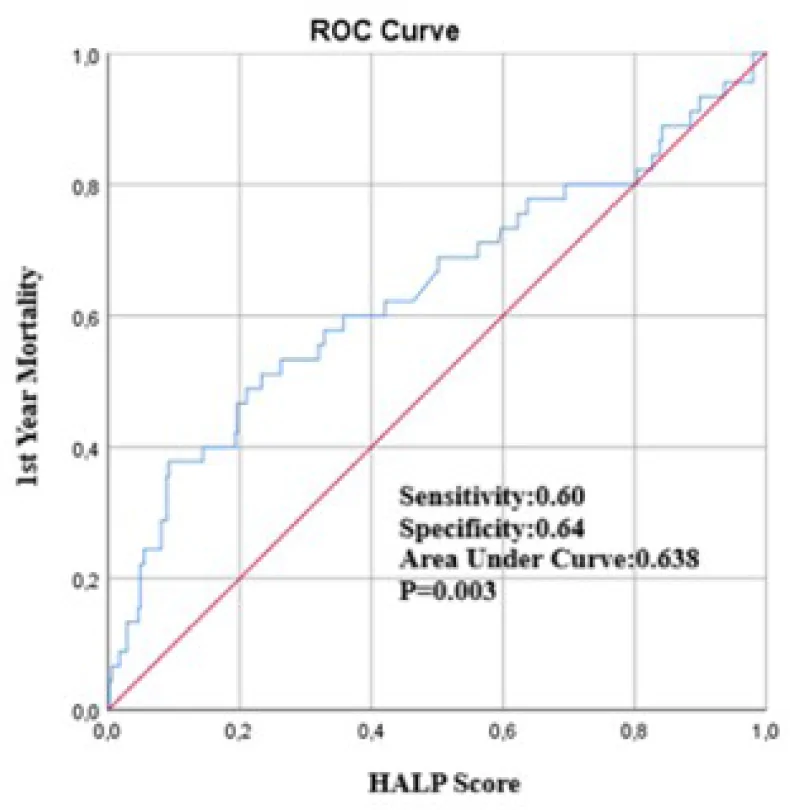
Receiver operating characteristic (ROC) curves showing the discriminative ability of the hemoglobin, albumin, lymphocyte, and platelet (HALP) score for one-year mortality. A HALP score cutoff of 3.4 was identified, with a sensitivity of 60% and specificity of 64% in patients undergoing transcatheter aortic valve implantation.

**Fig. 2:**
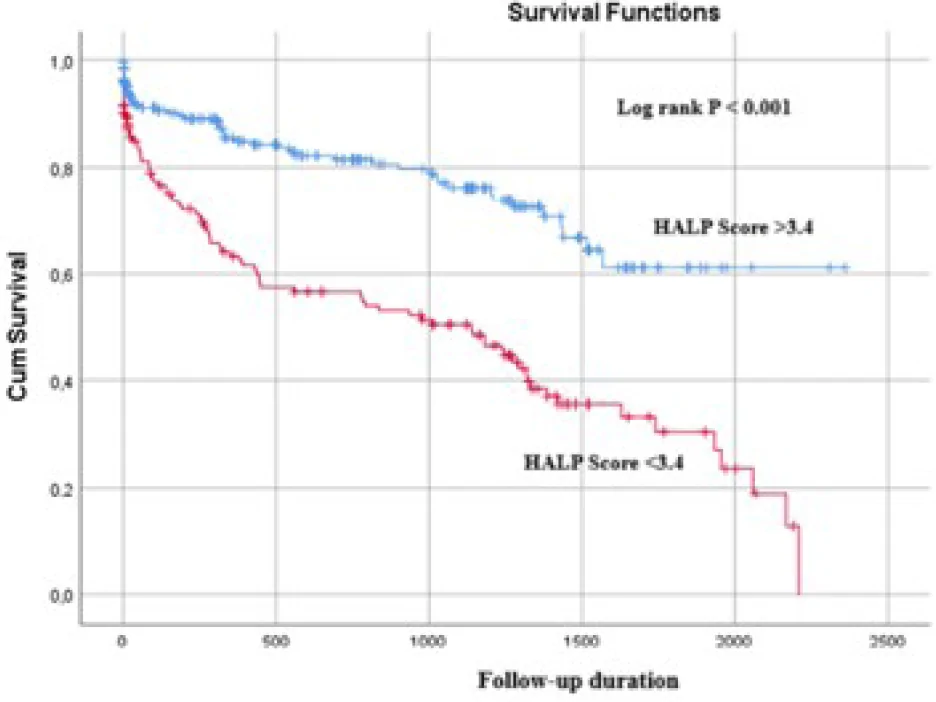
Kaplan-Meier survival curves showing significant differences in one-year all-cause mortality based on the hemoglobin, albumin, lymphocyte, and platelet (HALP) cutoff score of 3.4.

ROC analysis identified a HALP score cutoff of 3.4 for predicting one-year mortality in patients who have undergone TAVI, with a sensitivity of 60% and specificity of 64%. The area under the ROC curve was 0.638 (Figure 1). Kaplan-Meier survival analysis showed that patients with HALP scores below the cutoff value of 3.4 had higher mortality in the long-term follow-up, compared to those with higher HALP scores (Figure 2).

## DISCUSSION

In this study, we investigated the predictive value of the HALP score for one-year all-cause mortality and one-month MACE in patients undergoing TAVI. The results revealed that lower HALP scores were associated with increased all-cause mortality and MACE. Patients with lower HALP scores had poorer prognosis than those with higher scores, in the short term. To our knowledge, this is the first study to examine the HALP score as a prognostic tool for both one-month outcomes and one-year mortality in the TAVI population.

The HALP score is a composite biomarker that incorporates hemoglobin, albumin, lymphocytes, and platelet counts to reflect a patient’s nutritional and inflammatory status. Higher scores result from elevated serum hemoglobin, albumin, and lymphocyte levels and lower platelet counts. Low hemoglobin and albumin levels indicate anemia and malnutrition, whereas low lymphocyte counts and elevated platelet counts are associated with systemic inflammation and impaired immune function. Previous studies have identified the HALP score as a reliable predictor of long-term survival^[^13^]^ and a sensitive index for adverse outcome prediction, primarily in patients with malignancies^[^14^]^.

Despite the limited evidence on the association between the HALP score and prognosis in cardiology, the positive findings from studies conducted in the past two years may support its growing use in further research. Karakayali et al.^[^15^]^ hypothesized that a low HALP score could be a significant independent predictor of in-hospital mortality in patients with ST-segment elevation myocardial infarction (STEMI) treated with primary percutaneous coronary intervention (PCI). Similarly, Toprak et al.^[^16^]^ proposed that a low HALP score might independently predict the development of no-reflow and short-term mortality in patients with STEMI undergoing primary PCI. In line with previous studies, Zheng et al.^[^17^]^ demonstrated that a lower HALP score was associated with a higher risk of all-cause mortality, particularly in patients aged < 65 years, irrespective of congestive heart failure status. Furthermore, Liu et al.^[^18^]^ found that a lower HALP score may be a superior clinical predictor of outcomes in patients with heart failure.

Various hematologic parameters and indexes, including T-lymphocyte and monocyte subsets and the systemic immune-inflammation index (SII), have been investigated as short-term indicators of inflammation in patients undergoing TAVI. Increased intermediate monocyte levels and low baseline Th2 cell counts have been identified as significant predictors of mortality at six months and 12 months, respectively, in this population^[^19^,^20^]^. Consistent with these findings, SII, indicated by the platelet × neutrophil-to-lymphocyte ratio, was associated with six-month mortality in patients undergoing TAVI^[^21^]^. An elevated platelet-to-lymphocyte ratio, an indicator of inflammation and compromised immune function, has also been linked to 30-day adverse outcomes in patients undergoing TAVI^[^22^]^.

This study found a significant relationship between the HALP score and both one-month prognosis and one-year mortality in patients undergoing TAVI. Specifically, a low median HALP score was associated with an increased risk of stroke; VARC-3 type 2 - 4 bleeding; major vascular, access-related, or cardiac structural complications; stage 3 - 4 AKI; and new permanent pacemaker implantation at one month. The TAVI procedure itself induces considerable postprocedural systemic inflammation and oxidative stress^[^23^]^. We speculate that low albumin levels, which reduce the body’s available antioxidant and anti-inflammatory capacity, may contribute to postprocedural morbidity, including one-month MACE, especially in patients undergoing TAVI. In addition, low preprocedural albumin and hemoglobin/creatinine ratios, which are commonly observed in patients with CKD, have also been proposed as significant predictors of all-cause mortality following TAVI^[^24^,^25^]^. In this study, both a low HALP score (OR, 1.235; 95% CI, 1.091 - 1.397; *P* < .001) and CKD (OR, 4.67; 95% CI, 2.36 - 9.25; *P* < .001) were independent predictors of one-year mortality. The HALP score cutoff used in our analysis to predict one-year mortality was 3.4, which is similar to the cutoff of 3.72 identified by Karakayali et al.^[^15^]^ in patients with STEMI undergoing primary PCI. This discrepancy may be attributable to an increased platelet-to-lymphocyte ratio and decreased hemoglobin, albumin, and glomerular filtration rate levels observed in our study’s older population, compared with the cohort studied by Karakayali et al.^[^15^]^ Accordingly, consistent with previous studies, we suggest that an elevated platelet-to-lymphocyte ratio, as well as low hemoglobin and albumin levels, has a combined impact on patient outcomes.

The HALP score is a simple, practical index derived from routine blood markers and may provide a rapid, bedside approach to risk stratification, which can support individualized treatment and triage. As a readily calculable index derived from routine laboratory parameters, the HALP score may be used as a standalone risk marker or in conjunction with the STS score during the pre-intervention period. Early identification of high-risk patients using this score may allow clinicians to make more informed decisions regarding valve selection and to ensure closer postprocedural follow-up. Given its strong predictive value for morbidity and mortality, patients with HALP scores below 3.4 may warrant closer monitoring and follow-up after TAVI.

### Limitations

This study has some limitations. First, the relatively small sample size and single-center, retrospective design may limit the generalizability of the findings. Second, the HALP score is a dynamic marker that can fluctuate daily because of acute illness, hydration status, or transient inflammation. As such, a single preprocedural measurement may not adequately reflect a patient’s long-term nutritional or inflammatory status, potentially limiting its ability to predict short-term prognosis and mortality. Third, using a post-hoc cutoff value for the HALP score in ROC analysis may have reduced its prognostic strength in this study. Fourth, residual and unmeasured confounders may still exist, even after applying a multivariable model to account for possible variables. Finally, no formal frailty assessment (*e.g.*, gait speed or the Clinical Frailty Scale) was performed. Further studies in larger, more diverse cohorts are needed to confirm and improve its clinical utility.

## CONCLUSION

Our study is the first to demonstrate the predictive value of the HALP score for one-month prognosis and one-year mortality in patients undergoing TAVI. This study demonstrated that lower HALP scores were found to be an independent predictor of prognosis and mortality. In this context, this study clarified that the HALP score was a suitable marker for short-term risk stratification in the TAVI population.

## Data Availability

The authors declare that the data supporting the findings of this study are available within the article.

## Artificial Intelligence Usage

The authors declare that no artificial intelligence tool was used in the preparation of this article.
